# Tunable Optical Performances on a Periodic Array of Plasmonic Bowtie Nanoantennas with Hollow Cavities

**DOI:** 10.1186/s11671-016-1636-x

**Published:** 2016-09-20

**Authors:** Yuan-Fong Chou Chau, Chung-Ting Chou Chao, Jhin-Yu Rao, Hai-Pang Chiang, Chee Ming Lim, Ren Chong Lim, Nyuk Yoong Voo

**Affiliations:** 1 Centre for Advanced Material and Energy Sciences, Universiti Brunei Darussalam, Tungku Link, Gadong, BE1410 Negara Brunei Darussalam; 2 Department of Physics, Fu Jen Catholic University, New Taipei City, Taiwan; 3 Department of Electronic Engineering, Chien Hsin University of Science and Technology, No. 229, Jianxing Rd, Zhongli City, Taoyuan County 32097 Taiwan; 4 Institute of Optoelectronic Sciences, National Taiwan Ocean University, Keelung, 202 Taiwan; 5 Institute of Physics, Academia Sinica, Taipei, 11529 Taiwan

**Keywords:** Plasmonic bowtie nanoantennas, Finite element method, Hollow number, Cavity plasmon resonance

## Abstract

We propose a design method to tune the near-field intensities and absorption spectra of a periodic array of plasmonic bowtie nanoantennas (PBNAs) by introducing the hollow cavities inside the metal nanostructures. The numerical method is performed by finite element method that demonstrates the engineered hollow PBNAs can tune the optical spectrum in the range of 400–3000 nm. Simulation results show the hollow number is a key factor for enhancing the cavity plasmon resonance with respect to the hotspot region in PBNAs. The design efforts primarily concentrate on shifting the operation wavelength and enhancing the local fields by manipulating the filling dielectric medium, outline film thickness, and hollow number in PBNAs. Such characteristics indicate that the proposed hollow PBNAs can be a potential candidate for plasmonic enhancers and absorbers in multifunctional opto-electronic biosensors.

## Background

Broadband nanoantennas play a potential role in the nanophotonic field. Recently, plasmonic optical nanoantennas [1–4] made by novel metal nanoparticles (MNPs) have generated great research interest due to their capability or dramatically localizing and enhancing electromagnetic (EM) fields on the surface of the MNPs [5, 6] and have attracted much attention for near field applications in biosensing [7], spectroscopy [8], nanolithography [9, 10], etc. Among these traditional optical nanoantennas, plasmonic bowtie nanoantennas (PBNAs) [11, 12], which can efficiently convert light from free space into subwavelength scale with the local field enhancement at optical frequency in the small air gap between their two triangle MNPs. PBNAs are usually designed to induce high local EM fields in between the gap to be used in sensing applications.

To deeply exploit the surface plasmon resonance (SPR) effects on a periodic array of PBNAs, a fully understanding of the interaction between the incident EM wave and PBNAs is urgently needed. Optical transmittance measurements revealed that the PBNAs supported both the bonding and anti-bonding SPR modes, and finite element method (FEM) calculations show very high field intensities within the bowtie gap for the bonding SPR modes [13, 14]. Recently, absorber MNPs are also employed in the quantum dot solar cells to trap solar radiation in the form of localized SPRs, which lead to a broad spectral photocurrent enhancement [15]. In a gold nanofilm, strongly enhanced local field due to the excitation of SPRs will give rise to two-photon absorption and further lead to two-photon excite photoluminescence (TPPL). TPPL can be served as an efficient way to probe and image SPR modes in MNPs and resonant nanoantennas [16]. In the recent literatures, it was demonstrated that hollow PBNAs are capable of absorption radiation [1, 2, 17] and the operation wavelength is confined in the range of visible light. In fact, it should be considered that broadband resonances are commonly correlated to efficient absorption over the wider spectrum. Tuning the properties of PBNAs for the vast variety of applications relies heavily on a careful design approach which involves three major parameters: dimension, aspect ratio, and the inner/outer material in MNPs [18, 19]. The expanding application spectrum of PBNAs demand versatile design approaches to tailor the antenna properties for specific requirements.

The fabrication of the periodic array of hollow PBNAs based on secondary electron lithography was previously demonstrated [1, 2]. In fact, being hollow cavities, the PBNAs can work as an optical nanocavity that generates both outer and inner SPR modes along with the high hotspots exiting in hollow and gap regions far below the diffraction limit. This value is significantly high if we consider that the hollow works in nanoscale regime. It could open promising applications where high near-field intensity is needed in a free volume (air, gas, dielectric medium, or liquid) of subdiffraction dimension [1, 2]. In this paper, we numerically analyze and quantitatively compare the near-field intensities and absorption spectra in a periodic array of PBNAs by introducing the hollow cavities into the MNPs. Although several outline plasmonic nanoantenna structures have been proposed for tuning SPRs, for manipulating the damping of the plasmonic resonances, for achieving deep subwavelength light modulation, and for improving dielectric constant sensing capabilities [3, 20, 21], our study will demonstrate the significant freedom gained by a simple outline design to broaden and tune the SPR effects without changing the outer dimension of MNPs. We focused our attention on a period array of hollow PBNAs which show the accumulation of the optical energy in the hollow regions. Numerical simulations are performed by using three-dimensional (3-D) FEM [22]. We examine the influence of its structural parameters on the antenna resonance conditions (i.e., the effective resonant wavelength (*λ*_res_)). In addition, the characteristic of near-field intensities and absorption spectra of a periodic array of hollow PBNAs corresponding to their bonding mode has also been investigated.

## Methods

**Fig. 1:**
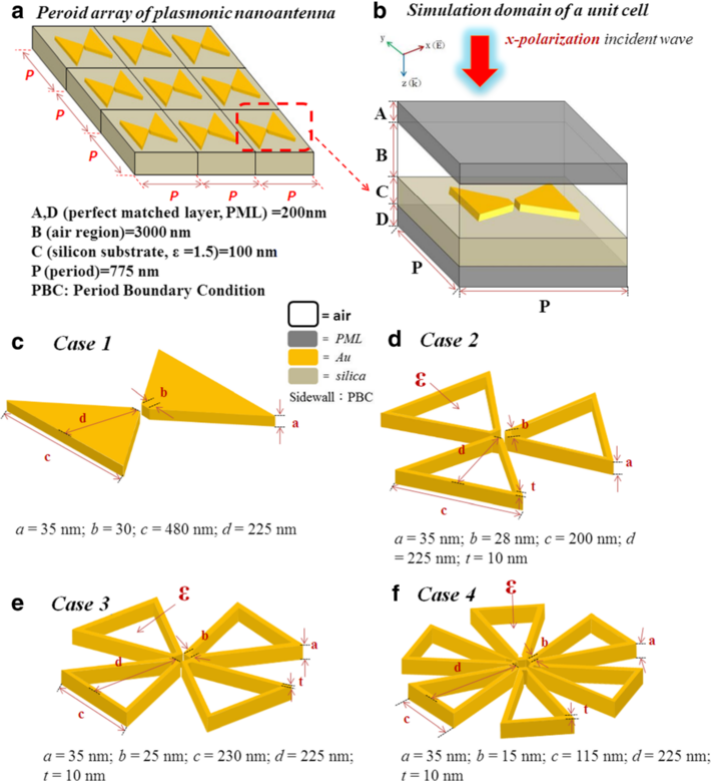
Schematic diagram of the simulation models. **a** A periodic array of plasmonic bowtie nanoantennas (PBNAs). **b** Simulation model of a unit cell. **c** Case 1, with a pair of bowtie-shaped MNPs. **d** Case 2, with three hollows in bowtie-shaped MNPs. **e** Case 3, with four hollows in bowtie-shaped MNPs. **f** Case 4, with six hollows in bowtie-shape MNPs. All simulation parameters are shown in the *inset* of this figure

The simulations are performed by using the 3-D FEM in the wavelength range of 400–3000 nm. Figure 1a shows the schematic diagram of a periodic array of PBNAs. For mimicking the structure of an infinite periodic array, the 2-D array of PBNAs was obtained from the unit cell by using periodic boundary condition (PBC) along the *x*- and *y*-axes and the anisotropic perfectly matched layers (PML) condition along the EM wave propagation direction. The unit cell of the traditional periodic array of PBNAs (see Fig. 1b) is composed of two gold triangular MNPs placed in close vicinity to each another, which can be characterized by four structure parameters (*a*, *b*, *c*, *d*) as shown in Fig. 1c: the antenna thickness, *a*; the apex width, *b*; the bowtie width, *c*; and the bowtie length, *d*. In addition, we also consider a periodic array of PBNAs by introducing hollow regions into the MNPs. The unit cell of the periodic array of PBNAs consists of three, four, and six metallic triangular outlines by six parameters (*a*, *b*, *c*, *d*, *t*, ε), as shown in Fig. 1d, e, and f, respectively, with additional structure parameters: the outline thickness, *t*, and the dielectric constant *ε* filled inside the hollow regions. The periodic array of PBNAs with period *P* along *x*- and *y*-directions are placed on a fused silica (*n*_s_ = 1.5) substrate normally illuminated with the *x*-polarization incident EM wave upper the substrate. The electric field amplitude of the incident light (│E_*i*_│), period (*P*), apex angle of antenna (*θ*), antenna thickness (*a*), outline thickness (*t*), gap width (*g*), apex width (*b*), and area of gap region (*A*) are set to be │E_*i*_│ = 1 V/m, *P* = 775 nm, *θ* = 90^0^, *a* = 35 nm, *t* = 10 nm, *g* = 30 nm, *b* = 30 nm and *A* = 30 × 30 nm^2^ throughout this paper unless otherwise specified. These parameter ranges have been chosen according to our previous works [13, 14] and extended calculations. The dielectric constants of gold were obtained from ref. [23].

## Results and discussions

**Fig. 2:**
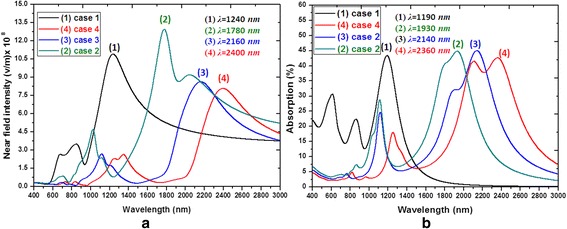
Comparison of **a** near**-**field intensities (measured in the gap region) and **b** absorption spectra of cases 1–4 in the range of incident wavelength of 400–3000 nm.

First, we compare the near-field intensities and absorption spectra of cases 1–4 in the incident wavelength range of 400–3000 nm as shown in Fig. 2a, b, respectively. The near-field intensities and absorption spectra show at least three peak values as a function of the wavelength of incident light, which correspond to different orders of Fabry-Perot resonances [2]. Cases 1–4 exhibit tunable performance regarding both the near field intensity and absorption. These behaviors can be related to the fact that the EM radiation can penetrate inside the hollow of PBNAs and show how broadband absorption can be converted to multiband enhancement at the nanometer scale. The absorption profile is due to the overlapping of many resonant modes which can be explained as: (1) low-order modes distributing on the outline surface of PBNAs; (2) high-order modes resonating inside the hollows and between the gap regions; (3) hybrid modes as due to a combination of the previous two, thus distributing on both inner hollow regions and outer outline surfaces of PNBAs [1, 2, 24].

Note that the *λ*_res_ of near-field intensity and absorption spectra shows a red shifting with the increasing hollow number (the number of triangular wings) in PBNAs due to the fact that more hollow regions result in enhancing the cavity plasmon resonance among them. These results in the splitting of the plasmon mode into two resonance modes, i.e., “bonding” mode (ranging in longer wavelengths) and “anti-bonding” mode (ranging in shorter wavelengths) [24]. The *λ*_res_ are found in the wavelength range of 1.2–2.5 μm for bonding mode and 0.6–1.3 μm for anti-bonding mode, respectively. The bonding mode occurs at longer wavelengths owing to the attractive near-field interactions across the gap and hollow regions lower than the resonant frequency [25]. Additionally, the more hollow number in PBNAs can contribute a change of *λ*_res_ toward longer wavelength with respect to the retarding effects among the MNPs. It is worthy to note that the advantage of case 4 is the polarization independence compared to the other cases with less hollow number. The *λ*_res_ of both bonding and anti-bonding modes can be easily tuned in the broad spectra range by adjusting their structure parameters, e.g., *a*, *b*, *c*, *d*, *t*, *ε*, and *P*, useful for various plasmonic optical sensing, solar cells, and optical device applications, owing to the coupling effects among neighboring gold-shell outline structures [13, 14]. This ability relies on the hollow cavity vertically aligned (with respect to the dielectric substrate plane) PBNAs. The multiband behavior exhibited by these hollow PBNAs may be a key factor for highly sensitive Raman measurements on the various components of the whole cell using an extended range of excitation light sources [1, 2].

**Fig. 3:**
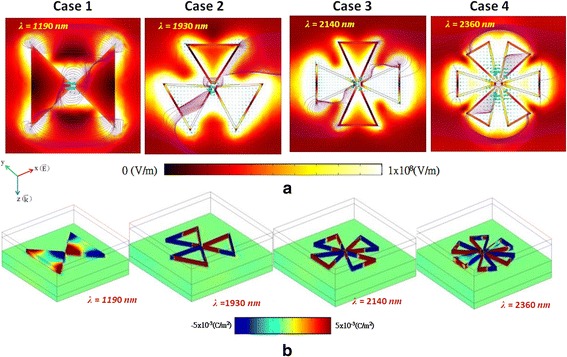
**a** Near-field distributions, energy flows (*cyan arrows*), and electrical filed stream lines (*purple lines*) corresponding to bonding modes (absorption spectra) for case 1 at *λ*_res_ = 1.19 μm, for case 2 at *λ*_res_ = 1.93 μm, for case 3 at *λ*_res_ = 2.14 μm, and for case *4* at *λ*_res_ = 2.36 μm, respectively. **b** Schematic charge densities corresponding to bonding modes of the cases 1–4

The field distributions, energy flows (cyan arrows), and electrical filed stream lines (purple lines) corresponding to bonding modes (absorption spectra) for case 1 at *λ*_res_ = 1.19 μm, for case 2 at *λ*_res_ = 1.93 μm, for case 3 at *λ*_res_ = 2.14 μm, and for case 4 at *λ*_res_ = 2.36 μm are shown in Fig. 3a, respectively. From these figures, we find that the electric field is enhanced on the edge surface of the hollow PBNAs and forms a hotspot within the gaps and hollow regions. The region of hotspot is increased as the increasing of the hollow number in PBNAs, which demonstrates that the cavity plasmon resonance contributes the SPR effects on PBNAs with hollow cavities.

The schematic charge densities of cases 1–4 are also depicted in Fig. 3b, respectively. In case 1, the charge pairs distribute locally on the edge surface of PBNAs, showing dipole-like distributions and resulting in bonding mode resonance. The charge pairs of cases 2–4 distribute overall on the inner/outer outline surface of PBNAs and exhibit stronger dipole-like distributions than that of case 1 due to the combination of SPRs and the cavity-like effects among the MNPs, gaps, and hollows. It is worthy to note that the segment of charge pair distribution is dependent on the hollow number being used in PBNAs. The mechanism can be explained by the symmetries and asymmetric modes of the charge pair distribution and the dipole and quadrupolar (or higher order) resonances of hollow PBNAs with/without central hollows or cavities [13, 14, 25–29].

**Fig. 4:**
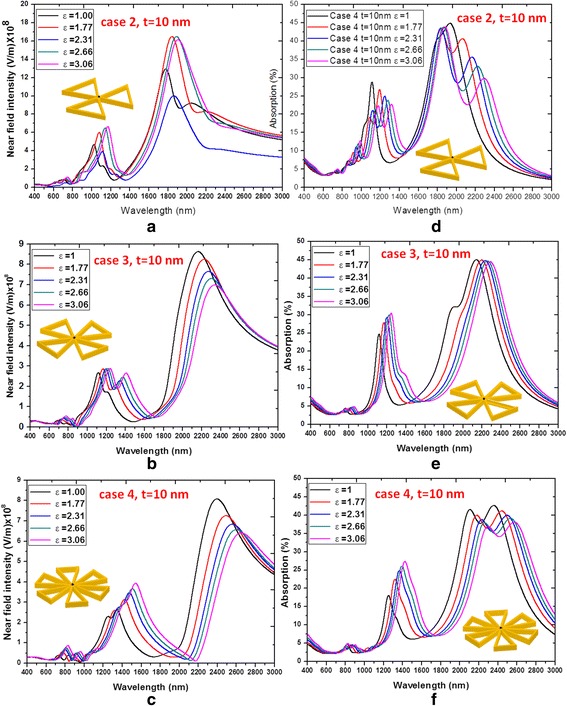
Behaviors of the near-field intensities (**a**–**c**) and absorption spectra (**d**–**f**) vs. wavelengths affected by the filling medium inside the hollow region of PBNAs with different *ε* (*ε* = 1.00, 1.77, 2.31, 2.66, and 3.06)

The detailed behaviors of the near-field intensities and absorption spectra vs. wavelengths affected by the filling dielectric constant inside the hollow region of PBNAs with different *ε* (*ε* = 1, 1.77, 2.31, 2.66 and 3.06) are shown in Fig. 4. The near-field intensities (see Fig. 4a–c) and absorption spectra (see Fig. 4d–f) show the same trend and have a red shifting with the increasing hollow number and an increasing dielectric constant, *ε*. Note that the family curves of Fig. 4a–f are quite different from the results obtained from Fig. 2a, b, showing multi-resonances behaviors in different wavelength range. Therefore, the hollow PBNAs with filling dielectric media (cases 2–4) can improve the sensor applications with its sensitivity of *ε* filled inside the hollow of PBNAs. The resonance characteristics of hollow PBNAs can be easily tuned by varying the filling medium, *ε*, and the geometrical parameters, *t* (will be discussed later). It can be seen from Fig. 4d–f that the resonance width of absorption is increased with the increasing hollow number in PBNAs. As a general rule, a broaden absorption resonance (i.e., broadband absorption) implies a more efficient electric field accumulation [29, 30]. This is not possible in traditional PBNAs, which usually presents only a few lower order resonances along the longitudinal and the transverse polarizations [13, 14, 31–39]. Therefore, the hollow regions in MNPs show a continuous transmission profile where individual resonances are independently excited. This behavior also results in a remarkable field enhancement over the whole spectrum range, as shown in Fig. 4a–c. Note that the gap enhancement factor (by calculating the near-field intensity measured in the gap and normalizing it to the spectrum incident EM wave) of case 4 (see Fig. 4c) is much higher than that of Fig. 4a, b.

**Fig. 5:**
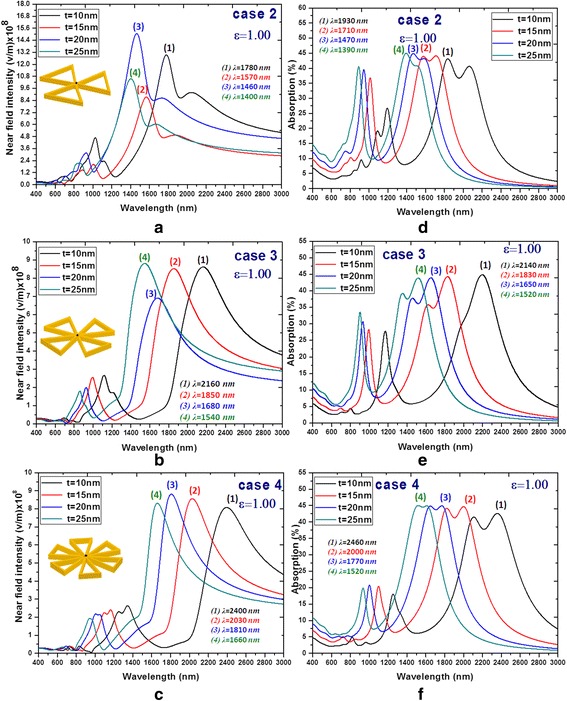
Behaviors of the near-field intensities (**a**–**c**) and absorption spectra (**d**–**f**) affected by the outline dimension of the hollow PBNAs with different outline film thickness *t* (*t* = 10, 15, 20, and 25 nm)

Similar to the filling dielectric constant, *ε*, the *λ*_res_ of hollow PBNAs is also dependent on the outline film thickness, *t*. From Fig. 5, a red shift of the operation bandwidth can be seen as the decreasing of outline film thickness *t*. The varying outline film thickness *t* would yield a change of the electron density, which is related to the number of positive-negative charge pairs [34]. This resonance shift enables deeper subwavelength control of light field by a hollow PBNA compared to a traditional PBNA of the same size. From Figs. 4 and 5, we can conclude that the coupling between these fields gives field distributions and enhancements on the surface of hollow PBNAs that are sensitive to the value of *ε* and outline film thickness *t*. It can be expected that the desired peak SPR values of near-field intensities and absorption spectra can be tuned by modifying ε and *t* in the full spectrum range of 400–3000 nm.

**Fig. 6:**
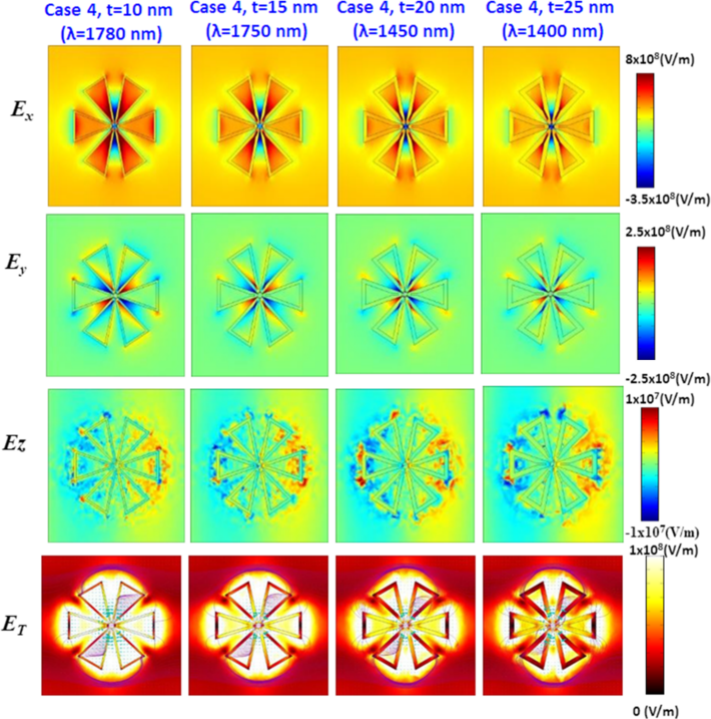
Near-field distributions of three electric filed components (*E*_*x*_, *E*_*y*_, and *E*_*z*_) and total field (*E*_total_) intensities at their corresponding *λ*_res_

To find the contribution of SPRs, we plot the near-field distributions of three electric field components (*E*_*x*_, *E*_*y*_, and *E*_*z*_) and total field (*E*_total_) intensities. The selected case 4 with different *t* (*t* = 10, 15, 20, and 25 nm) at their corresponding *λ*_res_ are investigated. From the results revealed in Fig. 6, *E*_*x*_ shows strong field distributions inside the central hollows, between the gaps, and around the outline surface of thin films. *E*_*y*_ is distributed symmetrically along the outline surface. *E*_*z*_ is weaker and shows a blurred near-field profile. *E*_total_ with electric stream lines (purple lines) show a hybridized plasmon mode on the inner and the outer outline thin film surfaces and gap regions. The strongest field intensity (peak value) is found within the bowtie gaps and hollow cavities. Note that the localized electric field enhancement at the circumference of the gold-shell PBNAs extends in the several tens of nanometers range from the PBNAs surface, which can be clearly seen from their electric stream lines. The efficient excitation of higher order modes leads to intriguing consequences from the point of view of tuning near-field intensities and absorption spectra. In the proposed hollow PBNAs, the electric field distributes entirely the hollow region; hence, when the medium, *ε* (gases or liquids), is flowed through the hollow region, they must experience the electric field action. Therefore, the hollow PBNA possesses the advantages of efficient excitation of high order modes that results in an effective broadband absorption and the capability of concentrating the electric and magnetic fields in the hollow regions.

**Fig. 7:**
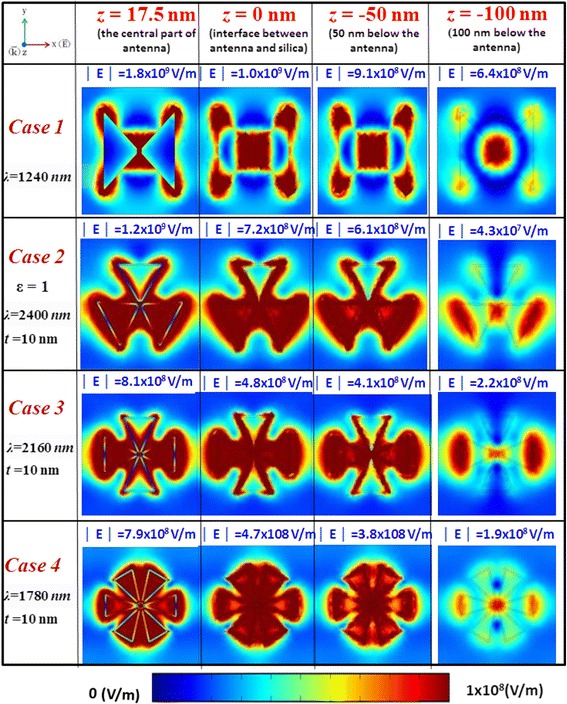
Distribution of electric field intensities corresponding to the bonding modes of cases 1–4 (from *top* to *bottom*) in the *x*-*y* plane measured at different planes along *z*-axis (from *left* to *right*), i.e., *z* = 17.5 nm (the central part of PBNA), *z* = 0 (the interface between PBNA and silica), *z* = −50 nm (a distance of 50 nm below the PBNA), and *z* = −100 nm (a distance of 100 nm below the PBNA), respectively. The electric field intensities (norm, in unit of V/m) are measured at the direction of midpoint along *z*-axis

Now, we quantitatively compare the difference of electric field intensities corresponding to the bonding modes of cases 1–4 in the *x*-*y* plane measured at different planes along *z*-axis as shown in Fig. 7, i.e., *z* = 17.5 nm (the central part of PBNAs), *z* = 0 (the interface between PBNAs and silica), *z* = −50 nm (a distance of 50 nm below the PBNAs), and *z* = −100 nm (a distance of 100 nm below the PBNAs), respectively. The electric field intensities are measured at the direction of midpoint along *z*-axis. It can be clearly seen from the first column of Fig. 7 that the electric fields at the central part of PBNAs are distributed among the gaps, hollows, and outline film surfaces corresponding to their bonding modes. For the selected case 4 as shown in the fourth row and first column of Fig. 7, the electric field intensity │E│ is enhanced up to =7.9 × 10^8^ V/m compared to the intensity of the incident light (│E_*i*_│ = 1 V/m). Meanwhile, the field patterns observed at a distance of 100 nm below the PBNAs (see the fourth row and fourth column of Fig. 7) show the same profile of PBNAs, and the field intensity is still as high as a magnitude of 1.9 × 10^8^ V/m. It can be found that the radiation wave still keeps a hotspot profile focusing on the gap region at a distance of 100 nm below the PBNAs.

## Conclusions

We numerically analyze and quantitatively compare the near-field intensities and absorption spectra of SPR modes on a periodic array of hollow PBNAs by means of 3-D FEM. We have found that the resonance width can be reduced as the increasing hollow number in PBNA due to the fact that more hollow regions result in enhancing the cavity plasmon resonance among them. It is worthy to note that the advantage of the proposed case 4 is the polarization independence compared to the other cases with less hollow number. The region of hotspot can be increased as the increasing of the hollow number in PBNAs, which demonstrates that the cavity plasmon resonance contributes the SPR effects on PBNAs with hollow cavities, showing the accumulation of the optical energy in the hollow regions. These promising results warrant a more detailed understanding of the performance of a periodic array of hollow PBNAs. The results shown in this work open new opportunities for design the broadband, flexible, and compact plasmonic devices for general purpose while the outer size of hollow PBNAs is kept constant.
